# Efficacy of cleansing agents in killing microorganisms in mixed species biofilms present on silicone facial prostheses—an in vitro study

**DOI:** 10.1007/s00784-015-1453-0

**Published:** 2015-04-07

**Authors:** Nina Ariani, Anita Visser, Margot R. I. M. Teulings, Melissa Dijk, Tri Budi W. Rahardjo, Arjan Vissink, Henny C. van der Mei

**Affiliations:** 1 University of Groningen and University Medical Center Groningen, Department of Biomedical Engineering, Antonius Deusinglaan 1, 9713 AV Groningen, Netherlands; 2 Department of Prosthodontics, Faculty of Dentistry, University of Indonesia, Jakarta, Indonesia; 3 University of Groningen and University Medical Center Groningen, Department of Oral Maxillofacial Surgery, Antonius Deusinglaan 1, 9713 AV Groningen, Netherlands

**Keywords:** Silicone elastomers, Facial prostheses, Biofilms, Mouthwashes, Chlorhexidine

## Abstract

**Objectives:**

The purpose of this study was to assess the efficacy of different cleansing agents in killing mixed species biofilms on silicone facial prostheses.

**Materials and methods:**

Two bacterial and three yeast strains, isolated from silicone facial prostheses, were selected for the mixed species biofilms. A variety of agents used to clean facial prostheses were employed, viz., antibacterial soap, essential-oil-containing mouth rinse, ethanol 27 %, chlorhexidine mouth rinse, and buttermilk. Colony forming units (CFUs) and live/dead staining were analyzed to assess the efficacy of these cleansing agents against 24-h and 2-week biofilms and regrown biofilms on silicone samples.

**Results:**

Chlorhexidine was the most effective cleansing agent. Chlorhexidine killed 8 log unit CFUs (>99.99 % killing) in a 24-h biofilm and 5 log unit CFUs (>99.99 % killing) in 2-week biofilms. Also, after regrowth and repeated treatment of the biofilm, chlorhexidine was the most effective cleansing agent showing no detectable CFUs. The essential-oil-containing mouth rinse (containing 26.9 % ethanol) showed a similar efficacy as ethanol (27 %) alone. Antibacterial soap and buttermilk were the least effective agents tested.

**Conclusions:**

Chlorhexidine showed the highest reduction in CFUs in 24-h, 2-week, and regrown mixed species biofilm of microorganisms isolated from silicone facial prostheses.

**Clinical relevance:**

Chlorhexidine mouth rinse (easy obtainable and relatively cheap) is very effective in killing bacteria and yeast present in biofilms on silicone facial prostheses. When applied on a regular basis, cleansing a facial prosthesis with chlorhexidine will presumably increase its lifetime and reduce skin irritations.

## Introduction

Patients suffering from facial defects caused by trauma, tumor removal, or congenital defects are often provided with facial prostheses made of silicone rubber in order to camouflage the defect. These facial prostheses have a limited lifetime of 1.5–2 years on average [1], however. This relatively short lifetime of facial prostheses is mainly caused by discoloration, deterioration of the prosthesis material by microbial ingrowth, material rupture, and aging [1–3]. These effects are due to the use of skin glue and exposure of the prostheses to environmental factors such as personal hygiene, environmental pollution (e.g., a dusty environment in a workshop), UV, temperature, and humidity [3–5]. Furthermore, skin secretions like perspiration and sebum [6, 7] and different cleansing treatments, such as using a microwave for prostheses without a clip construction and commercially available disinfectants, contribute to changes in the silicone rubber of which the prostheses are made [3, 8–10].

Silicone rubber facial prostheses can be retained using a variety of tools of which adhesives (skin glue) and dental implants are currently the most common ones [3]. Maintaining hygiene of the prosthesis is important for the health of the soft tissue underneath the prosthesis and for preserving the prosthesis itself in a good condition. Cleansing a facial prosthesis (with or without glue) or the skin (with or without an implant suprastructure) can be a difficult task, especially for patients with limited manual dexterity or visual problems, which is common in elderly who present the largest group amongst the facial prostheses wearers [1]. This is also reflected by the high prevalence of soft tissue infections around implants. Such skin reactions have been reported to occur in about half of the patients [1, 11]. Etiological factors like poor ventilation of the skin, accumulation of moisture, and compromised skin hygiene are presumed to be the most important factors causing skin irritations and infections [12, 13].

Acrylic resin and silicone facial prostheses may retain microorganisms, depending on the adhesion force with which these microorganisms adhere to the surface [7, 13, 14] and the cleansing skills of the patient [1]. The surface of the silicone prostheses can act as a reservoir for microorganism and yeast. Surface irregularities increase the possibility of harboring microorganisms, making the surface more difficult to cleanse [15, 16]. Mechanical methods, like brushing, have been shown to be insufficient to eliminate microorganisms colonizing acrylic resin dental prostheses [17]. Soft silicone materials used to reline dental prostheses are more difficult to cleanse than resins, and these soft materials are permeable and therefore susceptible to microbial colonization [15]. Fungal ingrowth was observed in nasal silicone prostheses and was associated with the black discoloration of these prostheses [18]. Black discoloration can also be caused by smoking [19].

Water and neutral soap, together with gentle brushing using a soft, nylon bristled toothbrush, are recommended for cleansing facial prostheses [14, 20] as well as the implants underneath the prosthesis. The use of chlorhexidine has been shown as an excellent auxiliary method to cleanse facial prostheses, along with the use of hydrogen peroxide and isopropyl alcohol [14, 20]. In an 18-month clinical longitudinal pilot study by Allen et al. [21] assessing the efficacy of a hygiene protocol for cleansing implant-retained facial prostheses, two thirds of the implant-retained facial prostheses had to be replaced due to silicone damage. Their pilot study as well as several other studies revealed that silicone rubber damage was caused by rigorous cleansing or use of inappropriate cleansing agents [8–10, 21, 22]. In line with this observation, a negative advice was given for mechanical cleansing of facial prostheses, e.g., by brushing. Repeated brushing also could contribute to discoloration of silicone rubber prostheses by dissolution and removal of surface pigments [8].

As far as we know, researchers did not yet investigate the efficacy of chemical cleansing with regard to killing of microorganisms that are present on silicone facial prostheses. A variety of cleansing agents has been used to cleanse silicone facial prostheses, the most common ones include soap, chlorhexidine, and isopropyl alcohol [14, 20], but the efficacy of these agents on killing of microorganisms present in mixed species biofilms on silicone facial prostheses was not yet studied. Therefore, the aim of the present study was to assess in vitro the efficacy of different cleansing agents in killing bacteria and yeast in a mixed species biofilm on silicone facial prostheses. The bacteria and yeast tested originate from mixed species biofilms that are present on used facial prostheses [13].

## Materials and methods

### Preparation of silicone samples

Silicone rubber (M511 Maxillofacial Silicone System, Technovent Ltd., South Wales, UK) commonly used to fabricate facial prostheses was used to make 60 × 60 × 1.5 mm sheets of silicone rubber. The silicone sheets were processed using plaster molds similar to molds used for processing facial prostheses. The silicone in the molds was polymerized with 5-bar pressure, at 45 °C for 90 min. To prevent adhesion of the silicone to the plaster molds, the surface of the molds was sprayed with releasing agent (MediMould, Polymed Limited, Cardiff, UK). After polymerization, the silicone was taken out from the mold. The technician wore new medical latex gloves to prevent adhesion of the skin flora onto the silicone sheets. To mimic the clinical situation, one side of the sheet was sealed with a silicone sealant (Multisil Sealant, Bredent, Senden, Germany) as used in clinical practice. Application of the sealant was according to the instructions of the manufacturer in order to reduce microbial colonization and therewith microbial penetration, preventing dirt adhesion, making it easier to cleanse, and improving adhesion to skin adhesives. Lastly, the silicone sheet was cut into samples of 15 × 15 mm with a sterile scalpel blade on a glass plate that was disinfected with 70 % ethanol. All silicone samples were sterilized by 70 % ethanol and air-dried under sterile conditions.

### Microbial strains, culture conditions, and biofilm formation

Two bacterial strains, *Staphylococcus epidermidis* MFP5-5 and *Staphylococcus xylosus* MFP28-3, and three yeast strains, *Candida albicans* MFP8, *Candida parapsilosis* MFP16-2, and *Candida famata* MFP29-1, were selected for the multi-species biofilms. All strains were retrieved from patients’ facial silicone prosthesis [13]. Each strain was grown on Brain Heart Infusion (BHI, OXOID, Basingstoke, UK) agar over night at 37 °C. One colony of amicrobial strain was inoculated in 5 mL of 30 % BHI and 70 % Yeast Nitrogen Base (YNB, BD Difco^™^, MD, USA; BHI/YNB) and incubated at 37 °C for 24 h. Subsequently, all strains were mixed in 1:1 volume ratio giving a multi-species suspension. The concentration of the bacterial culture was 2 × 10^9^/mL and yeast culture was 3 × 10^7^/mL.

A silicone sample was placed in each well of a 12-well plate (Costar, Corning, NY, USA). Six silicone samples were placed with the sealed side up and six with the sealed side down. The wells were inoculated with 2 mL of a multi-species suspension in BHI/YNB media and incubated for 3 h at 37 °C under aerobic condition for microbial adhesion. After 3-h incubation, the samples were washed with sterile phosphate buffer saline (PBS; 0.15 M NaCl and 10 mM potassium phosphate, pH 7.4) and moved to a new sterile well plate filled with 2 mL fresh BHI/YNB. The biofilm was allowed to grow for 24 h at 37 °C under aerobic conditions. All experiments were performed in triplicate.

To check whether a biofilm developed on the surface of the silicone samples after exposure to the selected bacterial and yeast strains, scanning electron microscopy (SEM) images were made on a regular basis. These SEM images revealed that that the surface of the silicone samples was covered with an extracellular matrix with bacteria and yeasts comparable to the biofilm present on SEM images of the surface of silicone facial prostheses [13].

### Treatment of biofilms with various cleansing agents

#### Twenty-four-hour-old biofilms

The following products were chosen: chlorhexidine and the essential oil products because they have good antimicrobial efficacies, ethanol 27 % in order to exclude that the ethanol in the essential oil product caused the killing of the microorganisms, buttermilk since it was very effective in reducing mixed species biofilm formation on silicone rubber voice prostheses, and antibacterial soap since soap is often advised for cleansing of silicone facial prosthesis.

After 24 h, the silicone rubber samples with biofilm were treated with one of the antimicrobial agents or cleansing solutions as mentioned in Table 1. This was done in order to measure the efficacy of the cleansing agents on killing microorganisms present in the biofilms. First, the samples in the 12-well plates were dipped once in water in order to remove the non-adhering microorganisms and subsequently immersed in 2 mL of the cleansing solution for 1 h at room temperature. Afterward, the samples were dipped once in water. The attached biofilm on the silicone samples was collected by swabbing with a sterile cotton swab stick and then suspended by vortexing in 1 mL sterile PBS.

**Table 1:** Antimicrobial agents and cleansing solutions used for treatment of the biofilm on silicone rubber samples

Antimicrobial agents/cleansing solution	Manufacturer	Active ingredients
Control Demineralized water		
Agents/cleansing solutions Demineralized water and Unicura Balance soap (1:1)	Colgate-Palmolive, Weesp, Netherlands	1–5 % cocamidopropylbetaine (surfactant/antiseptic), PPG-2-hydroxyethyl cocamide (surfactant), C12-16 pareth-7 (emulsifying, surfactant), cacamide MEA (foaming agent, surfactant), triclocarban (antibacterial, antifungal), laureth-4 (surfactant, emulsifier)
Listerine Original	Johnson & Johnson Consumer, Maidenhead, UK	0.092 % eucalyptol, 0.06 % methyl salicylate, 0.064 % thymol, 0.042 % menthol
Ethanol 27 %^a^		
Corsodyl mouthwash	GlaxoSmithKline Consumer Healthcare BV, Zeist, The Netherlands	0.2 % chlorhexidinedigluconate
Buttermilk	Friesland Campina, Amersfoort, Netherlands	

^a^Ethanol percentage in Listerine Original is 26.9 %

The suspended biofilms were serially diluted, and 100 μL of each dilution was plated on BHI agar plates and incubated at 37 °C under aerobic conditions for 24 h before colony forming units (CFUs) were counted. The detection limit with this method is 10–20 CFUs/mL. The suspended biofilms were also stained for 15 min with live/dead stain (1:1) (*Bac*Light^™^, Invitrogen, Breda, Netherlands), and the percentage of dead bacteria and yeast was determined. Three images along the sample were taken using a Leica DM4000B Fluorescence Microscope (Leica Microsystems Heidelberg GmbH, Heidelberg, Germany). Note that live/dead staining is not a measure of microbial killing but of membrane damage [23–25]. The membrane of live microorganisms is permeable to SYTO9, staining both live and dead microorganisms and yielding green fluorescence. Propidium iodide can only enter through damaged membranes, where it replaces SYTO9, yielding red fluorescence of dead or damaged cells.

#### Two-week-old biofilms

To check the efficacy of cleansing agents on more mature biofilms, biofilms were grown on silicone samples for 2 weeks. The biofilms were grown as above except that the biofilm was allowed to grow for 2 weeks. The growth medium was refreshed every second day. At day 14, the biofilms were treated and the biofilm samples were collected by swabbing and were suspended by vortexing in 1 mL sterile PBS for plating and fluorescence microscopy as described above. A 2-week biofilm was tested in order to determine whether a patient can start every moment with the cleansing procedure or that it is only effective for killing microorganisms in young biofilms.

#### Regrowth of a treated biofilm

Immediately after removing the 24-h treated biofilm on the silicone samples, the samples were placed in a 12-well plate with the biofilm side up. The wells were filled with 2 mL BHI/YNB growth medium and incubated for another 24 h at 37 °C. This procedure mimics the daily use of the prostheses. After incubation for 24 h, the biofilms were treated with the same cleansing solutions as before and biofilms were analyzed with the same methods as mentioned above to study repeated exposures to cleansing agents. This regimen was used to mimic the efficacy of repeatable cleansing of a facial prosthesis by the patient.

### Determination of the MIC and MBC

For the most promising cleansing agents, the minimal inhibitory concentration (MIC) and minimal inhibitory bactericidal concentration (MBC) per microbial strain were determined. A microbial suspension in growth media (5 · 10^4^ microorganisms/mL) was incubated together with serially diluted cleansing agents in a 96-well plate for 24 h at 37 °C. The microbial suspension with the cleansing agent which did not show any growth was determined as the MIC. The clear suspensions were plated on agar, and when there was no growth, this was determined as the MBC.

### Statistical analysis

Two-tailed *t* test on the log units of CFUs was used to detect differences between the different biofilm and treatment groups. A significance level of *p* < 0.05 was used. Note that the sealed and unsealed sides of the silicone samples were taken together resulting in *n* = 6 for statistical analyses.

## Results

No statistically significant differences in CFU counts of the mixed species biofilms were observed between data derived from sealed and unsealed sides of the silicone samples treated with the same cleansing agents. Therefore, data from sealed and unsealed sides of the silicone were combined for each cleansing agent.

**Fig. 1:**
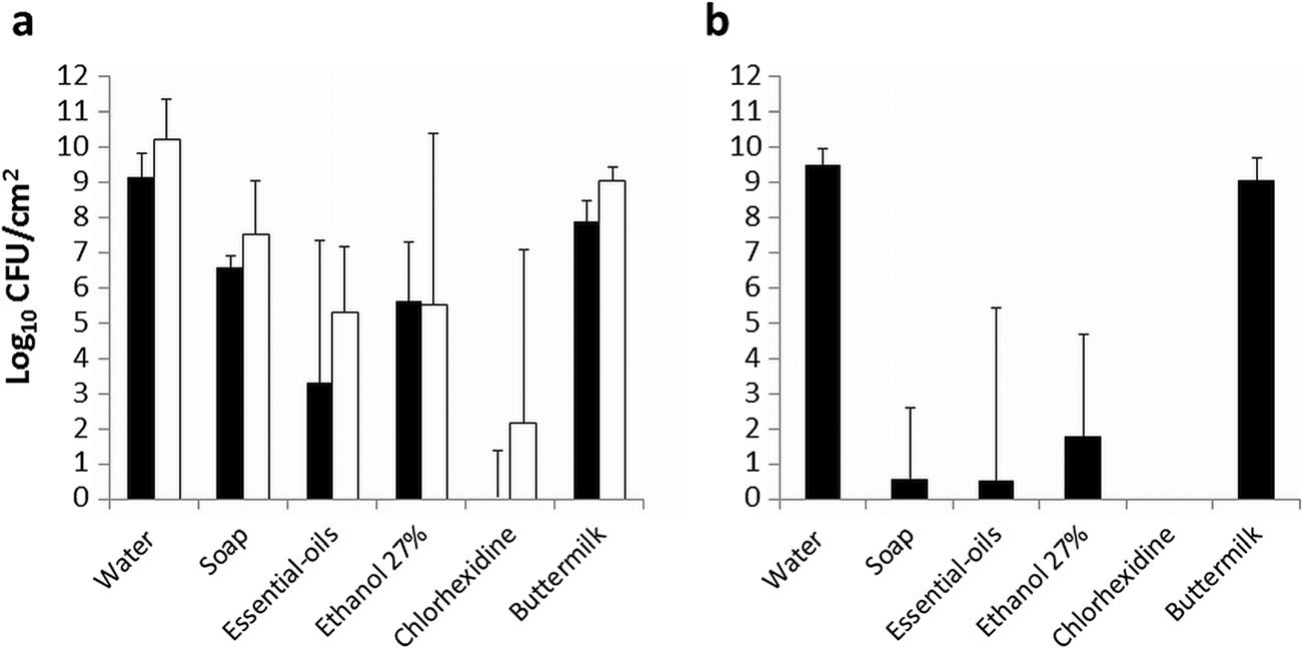
CFU of mixed species biofilms on silicone rubber after treatment with different cleansing agents. **a** Log CFU/cm^2^ of mixed species 24-h (*black boxes*) and 2-week (*white boxes*) biofilms on silicone rubber samples after treatment with different cleansing agents and water as a control. **b** Log CFU/cm^2^ of mixed species biofilm after treatment with various cleansing agents of a regrown 24-h mixed species biofilm. For statistical significances, see Table 2

When comparing the various cleansing agents with the control (water), all cleansing agents were significantly more effective than the control (Fig. 1). Chlorhexidine was the most and buttermilk the least effective cleansing agent for all time points studied (Fig. 1). The essential oil product (containing 26.9 % ethanol) showed a similar efficacy than when only the ethanol 27 % was tested (Table 2). All cleansing agents were less effective for a 2-week-old biofilm compared to a 24-h biofilm. Chlorhexidine killed 8 log unit CFUs in a 24-h biofilm, whereas in a more mature biofilm (2 week), 6 log unit CFU reduction was observed (Fig. 1). After treatment, the regrowth of the treated 24-h biofilm showed a high efficacy for the antibacterial soap, essential oil product, ethanol 27 %, and chlorhexidine (Fig. 1). Chlorhexidine killed all microorganisms and no growth was detectable. Note that the regrown biofilm has been treated two times, directly after the 24-h growth period and again after the regrowth of the treated biofilm.

**Table 2:** Between-group significance level of CFU counts after exposure of 24-h, 2-week, and regrowth of biofilms to different cleansing agents (*n* = 6)

Time	Agents	Soap	Essential-oils	Ethanol 27 %	Chlorhexidine	Buttermilk
24 h	Water	0.000*	0.005*	0.007*	0.000*	0.008*
	Soap		0.067	0.269	0.001*	0.002*
	Essential oils			0.257	0.060	0.016*
	Ethanol 27 %				0.003*	0.036*
	Chlorhexidine					0.000*
2 weeks	Water	0.007*	0.001*	0.031*	0.001*	0.054
	Soap		0.051	0.414	0.019*	0.055
	Essential oils			0.582	0.239	0.004*
	Ethanol 27 %				0.169	0.091
	Chlorhexidine					0.005*
Regrowth of biofilms	Water	0.000*	0.001*	0.019*	0.000*	0.211
	Soap		0.686	0.449	0.083	0.000*
	Essential oils			0.666	0.080	0.002*
	Ethanol 27 %				0.105	0.024*
	Chlorhexidine					0.000*

*p < 0.05

**Fig. 2:**
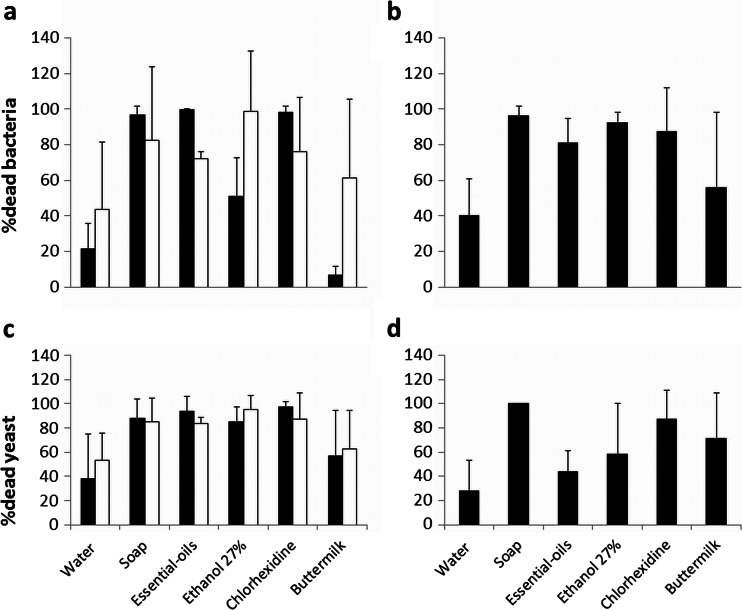
Mean and standard deviations of %dead microorganisms in mixed species biofilms on silicone samples that are treated with different cleansing agents compared to water (control). %dead bacteria of 24-h (*black boxes*) and 2-week (*white boxes*) biofilms. **a** %dead bacteria of 24-h (*black boxes*) and 2-week (*white boxes*) biofilms. **b** %dead bacteria of regrowth of biofilms after treatment. **c** %dead yeast of 24-h (*black boxes*) and 2-week (*white boxes*) biofilms. **d** %dead yeast of regrowth of biofilms following exposure to different cleansing agents. For statistical significances, see Tables 3 and 4

In Fig. 2, the %dead bacteria and yeast is presented. Soap, ethanol, essential oil product, and chlorhexidine were significantly more effective than the control in killing bacteria and yeast for 24-h and 2-week-old biofilms. Only the buttermilk was not significantly different from control (water). Tables 3 and 4 depict the efficacy of the various cleansing agents in killing bacteria and yeast.

**Table 3:** Between-group significance level of %dead bacteria after exposure of 24-h, 2-week, and regrowth of biofilms to different cleansing agents (*n* = 6)

Time	Agents	Soap	Essential-oils	Ethanol 27 %	Chlorhexidine	Buttermilk
24 h	Water	0.000*	0.000*	0.000*	0.000*	0.003*
	Soap		0.038*	0.000*	0.460	0.000*
	Essential oils			0.000*	0.016*	0.000*
	Ethanol 27 %				0.000*	0.000*
	Chlorhexidine					0.000*
2 weeks	Water	0.000*	0.030*	0.000*	0.015*	0.217
	Soap		0.063	0.053	0.143	0.018*
	Essential oils			0.007*	0.752	0.447
	Ethanol 27 %				0.022*	0.003*
	Chlorhexidine					0.298
Regrowth of biofilms	Water	0.001*	0.004*	0.001*	0.001*	0.255
	Soap		0.063	0.204	0.252	0.001*
	Essential-oils			0.140	0.493	0.049*
	Ethanol 27 %				0.529	0.003*
	Chlorhexidine					0.017*

*p < 0.05

**Table 4:** Between groups significance level of %dead yeast after exposure of 24-h, 2-week, and regrowth of biofilms to different cleansing agents (*n* = 6)

Time	Agents	Soap	Essential-oils	Ethanol 27 %	Chlorhexidine	Buttermilk
24 h	Water	0.002*	0.001*	0.003*	0.001*	0.265
	Soap		0.271	0.494	0.037*	0.017*
	Essential oils			0.040*	0.162	0.007*
	Ethanol 27 %				0.001*	0.029*
	Chlorhexidine					0.004*
2 weeks	Water	0.000*	0.000*	0.000*	0.000*	0.263
	Soap		0.322	0.067	0.725	0.002*
	Essential oils			0.016*	0.456	0.017*
	Ethanol 27 %				0.017*	0.000*
	Chlorhexidine					0.004*
Regrowth of biofilms	Water	0.000*	0.226	0.125	0.000*	0.004*
	Soap		0.007*	0.040*	0.095	0.018*
	Essential oils			0.440	0.005*	0.064
	Ethanol 27 %				0.130	0.508
	Chlorhexidine					0.213

*p < 0.05

The MIC and MBC were determined for chlorhexidine, essential oil product, and 27 % ethanol. For all strains tested in this study, the MIC and MBC of chlorhexidine was 0.06 % chlorhexidine digluconate (30 times dilution of the chlorhexidine product). The essential oil product could be diluted up to four times (corresponding to 6.7 % ethanol) for the MIC for all strains, except for *C. albicans* where the MIC was a two times (corresponding with 13.5 % ethanol) dilution of the product. The MBC was a two times dilution of the essential oil product for all strains. The MIC and MBC of ethanol were 27 % for all strains. Note that the MIC and MBC were tested on single strains which can explain the difference in behavior between the essential oil product and 27 % ethanol.

## Discussion

In this study, the efficacy of cleansing agents on silicone used for silicone facial prostheses was tested on their ability to kill mixed species biofilms. All tested cleansing agents proved to be more effective than control (water) in killing bacteria and yeast that were present in 24-h- and 2-week-old mixed species biofilms as well as in double-treated mixed species biofilms. Chlorhexidine seems to be very effective, while buttermilk was shown to be the least effective agent in killing microorganisms. The latter might be due to the bacteria present in the buttermilk used in this study, which bacteria also grew on agar plates.

No cultivable biofilm was present on the regrowth and double-treated biofilm on silicone samples after treatment with chlorhexidine. Chlorhexidine is a widely used antiseptic agent for prevention of biofilm formation and also promotes removal of biofilms of, e.g., *S. epidermidis*, *C. albicans*, and *C. parapsilosis* [26–28]. Chlorhexidine was less effective in killing microorganisms in a 2-week-old mixed species biofilm but was still the most effective of the tested cleansing agents in this study. The fact that cleansing agents become less effective for matured biofilms is not surprising as aged biofilms have been shown to possess more resistance to antimicrobials than young biofilms [29–31]. Matured biofilm is embedded in a polysaccharide matrix which reduces penetration of antimicrobials through the biofilm [32]. Other means of how biofilms develop resistance to antimicrobials are changes in the chemical environment within the biofilm that produce zones of slow or no growth, adaptive stress responses, and presence of persister cells [32–34]. Some facial prosthodontists, on basis of their experience, already advise their patients to clean their facial prosthesis with chlorhexidine, although there was yet no evidence that this product would be effective for killing the biofilm. This study showed that chlorhexidine is indeed effective in killing microorganisms present in the mixed species biofilm on silicone facial prostheses.

Essential-oil-containing mouth rinse, although not as effective as chlorhexidine, is frequently used to reduce the presence of (potentially) pathogenic microorganisms present in oral biofilms [35, 36]. The essential oils showed a similar efficacy in killing microorganisms than ethanol 27 %, showing no additional effect of the essential oils. This was observed earlier in a gingivitis study where the essential oil product was compared with ethanol [37]. The only difference we observed, between ethanol and essential oils, was that ethanol alone had a higher MIC and MBC than essential oil product. MIC and MBC were tested on single strain bacteria which can explain the fact that there was a difference observed. However, essential oils have been shown to be effective as an adjunct to mechanical biofilm removal as well [38]. Netuschil et al. [39] showed that essential oils work best against young and sparse oral biofilms as was also observed in this study for 24-h biofilms [39].

Buttermilk was involved as one of the cleansing agents which might be effective for cleansing silicone facial prostheses as buttermilk has been shown to be very effective in reducing mixed species biofilm formation on silicone rubber voice prostheses [40, 41]. However, buttermilk was not very effective in killing microorganisms present in the biofilm on facial prostheses, however. In the present study, we only did a single treatment with buttermilk on the biofilm, while in the voice prosthesis study, the biofilm in an artificial throat device was perfused with buttermilk three times a day for 9 days [42]. Our study was designed to study the effect of a single exposure of a biofilm to a cleansing agent, so we cannot exclude that buttermilk is effective in killing the studied biofilms when repeatedly exposed to buttermilk. Note that there are two types of buttermilk available in the Netherlands—one contains *Lactococcus lactis* and *Lactococcus cremoris* and the other one is pasteurized buttermilk which contains no viable bacteria. Only buttermilk containing viable *L. lactis* and *L. cremoris* was shown to reduce yeast colonization [42]. Our study used unpasteurized buttermilk, thus buttermilk containing viable bacteria.

The sealant that was applied on one side of the silicone samples was shown to be not effective in preventing microbial colonization of the samples. Although the sealant lowers the surface roughness of the silicone materials, other factors important for biofilms growth, such as nutrients and temperature, were still providing an environment for the microorganisms overpowering the effect of surface modification by the sealant. Further study is needed to confirm the presumed other properties of sealant such as preventing dirt adhesion and improving adhesion to skin adhesives as the sealant did not prevent microbial colonization of the samples.

CFU results showed that the number of microorganisms was lower after regrowth compared to 24-h and 2-week biofilm, except for water and buttermilk. The CFU counts for the 2-week biofilms were higher than for the 24-h biofilm, because in 2 weeks, biofilms have developed resistance [29]. Thus, the patient has to repeat the cleansing of their facial prosthesis to potentially become effective as possibly some biofilm might reside in niches on the prosthesis. Like for toothbrushing, repeated cleansing is the most effective means of proper cleansing all spots while cleansing with too long intervals will render in a less effective cleansing action as the biofilms have become more resistant [43, 44].

This study adds to the knowledge of how to maintain the facial prosthesis clinically in a good condition and avoid possible skin irritations, which advices are currently mainly based on the experience of maxillofacial prosthodontists who fabricate such prostheses. Clinicians working with patients needing facial prosthodontics have suggested the patients to soak their prosthesis in the mouth rinse with essential oils, believing that it helps to reduce skin irritation underneath the prosthesis (personal communication). Our results confirmed their suggestion. In addition, our results also showed efficacy of other cleansing agents that might help patients maintaining health of the skin covered by the facial prosthesis as well as preserving the prosthesis itself. For good antimicrobial efficacy, a minimum of 3 log reduction in CFUs is advised, showing that the essential oil product and chlorhexidine are both good choices for patients to use for cleaning of their facial prostheses. Taking both published recommendations on silicone facial prostheses maintenance [14, 19] and the results of our study into account, we propose the following maintenance regimen to be advised to patients to prolong the lifetime of silicone facial prosthesis and to be an asset in reducing the skin problems that occur beneath facial prostheses: Cleanse a silicone facial prosthesis by soaking the prosthesis in one of the cleansing agents that were shown to be effective in this study, preferably chlorhexidine. This procedure has to be repeated on a daily basis to achieve best prosthesis hygiene and to reduce skin irritation caused by microorganisms.
